# Unraveling the venom chemistry with evidence for histamine as key regulator in the envenomation by caterpillar *Automeris zaruma*

**DOI:** 10.3389/fimmu.2022.972442

**Published:** 2022-08-24

**Authors:** Andrea Seldeslachts, Steve Peigneur, Dietrich Mebs, Jan Tytgat

**Affiliations:** ^1^ Toxicology and Pharmacology, KU Leuven, Leuven, Belgium; ^2^ Institute of Legal Medicine, Goethe University Frankfurt, Frankfurt, Germany

**Keywords:** *Automeris zaruma*, caterpillar venom, histamine, histamine receptors, MRGPRX2 receptor, electrophysiology, Q-TOF-MS, antihistaminic drugs

## Abstract

Over the past decades, envenomation by caterpillars of *Automeris* spp. became an increasing health problem in Latin America. Accidental contact with the stinging spines of these caterpillars cause acute local pain, itching, inflammation and skin rashes that persists for days. Even when the cause is obvious, the exact molecular mechanisms responsible for the observed symptoms are yet to be elucidated. Here, we describe for the first time, an active compound in the venom and the study of the bioactivity of the venom extracted from the spines of the caterpillar *Automeris zaruma*. Electrophysiological screening of a library of membrane proteins important for pain and itch enabled us to investigate and reveal the mode of action of the venom of *A. zaruma*. Further mass spectrometric analysis (Q-TOF-MS) made it possible to establish a link between the bioactivity and the components found in the venom. We show that the spine extract of *A. zaruma* contains histamine that potently activates the four types of the human histamine receptors (H1R, H2R, H3R and H4R) with a selectivity preference towards H3R and H4R. Furthermore, a modulation of the target MRGPRX2 was found. Together, these findings are the first to explain the symptomology of *A. zaruma* envenomation, enabling us a better understanding of caterpillar envenomation and predict that the hurdle of the scarce efficacy of the currently used antihistaminic drugs can be overcome by including H3R and H4R blockers in the clinical used medication. Such an approach might be used for other caterpillar envenomation in the world and represent a significant improvement for the well-being of the patient.

## 1 Introduction

Caterpillar envenomation is still an underestimated and neglected public health issue with long-term negative social, economic and environmental impacts. Nine of the 133 caterpillar families in the order Lepidoptera are considered highly venomous for humans and animals (1, 2). In this respect, members of the Saturniidae family such as *Automeris* species are of special interest (3). These caterpillars occur in Latin America from Mexico, Ecuador to Argentina, and are recognized for their large size, bristle and colorful appearance. Contact with their spines result in a painful stinging sensation within seconds of exposure, followed by itching, skin rashes and erythema that may last for days (4). The needle-shaped spines of the integument are filled with a mixture of bioactive compounds. Upon contact with the skin, the tip of the spines breaks off and venom is injected (5).

Despite strong skin irritation and local pain, most of the cases do not require hospitalization. Nowadays, only supportive treatments are recommended because target therapies are still not available (1). This is mainly due to the fact that the number of caterpillar species studied remains extremely low compared to multiple studies performed on the content and interaction of venoms from snakes, spiders and scorpions. Venoms are known to carry a treasure of toxins/proteins/peptides that are evolved to interfere with high efficacy and potency for physiological targets in their victim’s core system to protect themselves against predators (1).

To date, there exist numerous studies on the venom composition of *Lonomia* and *Hylesia* caterpillars and some more comprehensive studies show the effect of *Latoia consocia* venom acting on ion channels (6–9). Unfortunately, little is known about the cutaneous effects of *Automeris* caterpillars (10). Moreover, the venom composition and venom bioactivity are still unknown. But, the immediate discomfort after contact with the spines may suggest the presence of small-molecular compounds such as acetylcholine, prostaglandins, and/or kinins (10).

Hence, in the present study, the venom chemistry and bio-activity of *A. zaruma* are described. The venom effect was examined electrophysiologically using a library of membrane proteins involved in itch, inflammation and pain. As such, we were able to explore the toxicity of the caterpillar *A. zaruma* venom and pinpoint important molecular targets. Together with the recent advances in cutting-edge technologies such as proteomics, the venom of *A. zaruma* is further quantified and qualified. In this way, the causality between substances found in the venom and some clinical symptoms seen in humans is unraveled. Altogether, these results form the foundation for a better understanding of *A. zaruma* venom and point towards an adequate treatment to tackle the envenomation.

## 2 Materials and methods

### 2.1 Collection, culture and venom harvesting

Over the course of this research, *A. zaruma* eggs were obtained from private breeders. At ambient temperature (about 20°C), the caterpillars were reared on oak leaves (*Quercus* spp.). For venom collection, 14 larvae (L5 stage) were frozen at -20°C, the dorsal and subdorsal spines were cut with scissor and lyophilized. Between 30-50 mg spines were combined, suspended in 50% acetonitrile (ACN), homogenized by manually crushing with a mortar and/or in a bead mill (Retsch MM 400, 30.0 Hz, 15 min) and cleared by centrifugation (12,500 rpm, 10 min). After centrifugation, the resulting supernatant was lyophilized and used for analysis.

### 2.2 Scanning electron microscopy (SEM)

The stinging spines of *A. zaruma* were mounted on an aluminum holder, sputtered with gold and analyzed with a Hitachi S-4500 scanning electron microscope at an acceleration voltage of 5 kV (cold-field emission electron source).

### 2.3 *Xenopus laevis* frogs

All animal experiments were approved by the KU Leuven Ethical Committee for animal research (Project No. P186/2019). Frogs are kept in aquatic tanks in the Aquatic Facility of KU Leuven according to the regulation and procedures agreed with the guidelines of the European Union (EU) concerning the welfare of laboratory animals, as declared in Directive 210/63/EU.

### 2.4 Isolation of *Xenopus laevis* oocytes by partial ovariectomy

Oocytes of stage V-VI were harvested from an anesthetized female *Xenopus laevis* frog by partial ovariectomy. Frogs were anesthetized by immersing in a solution of tricaine (ethyl 3-aminobenzoate methanesulfonate, 1 g/L, Sigma-Aldrich, USA) and NaHCO_3_ (sodium bicarbonate, 1 g/L; Sigma-Aldrich, USA) in aquarium water (pH 7.5) for 15 minutes. The isolated ovarian lobes were enzymatically defolliculated in a calcium-free ND96 solution (96 mM NaCl (Merck, Germany), 2 mM KCl (AppliChem GmbH, Germany), 2 mM MgCl_2_ (Merck, Germany) and 5 mM HEPES (Acros Oganics, Belgium) supplemented with collagenase from *Clostridium histolyticum* type IA (1.5 mg/mL; Sigma-Aldrich, USA) on a rocker platform at 16°C. Oocytes were then transferred and maintained into a calcium-containing ND96 solution (96 mM NaCl, 2 mM MgCl_2_, 2 mM KCl, 5 mM HEPES and 1.8 mM CaCl_2_ with a pH of 7.5) supplemented with geomycine (100 mg/L; Schering-Plough, Belgium) and theophylline (90 mg/L; ABC chemicals, Belgium) at 16°C.

### 2.5 *In vitro* synthesis of messenger RNA and injection

In the present work, different types of ion channels and receptors were investigated (1): voltage-gated sodium channels (rNa_v_1.2, rNa_v_1.3, rNa_v_1.4, mNa_v_1.6 and hNa_v_1.8) and their auxiliary subunits rβ1 and hβ1 (2), potassium channels (hK_v_1.1, hK_v_1.2, hK_v_1.3, hK_v_11.1 or hERG, dm*Shaker*-IR, mGIRK1, mGIRK2 and mIRK1) (3), histamine receptors (hH1R, hH2R, hH3R and hH4R) (4) bradykinin receptor (hB2R) (5), cannabinoid receptor (hCB2R) (6), Mas-related G protein-coupled receptor member X2 (hMRGPRX2) (7), transient receptor potential channel (hTRPV1) (8), muscle-type nAChR (hα1β1γδ) (9), neuronal-type nAChR (hα7) and (10) acid-sensing ion channel (rASIC1a). For the heterologous expression in oocytes, the plasmids for each channel/receptor were linearized using appropriate restriction enzymes and were transcribed using the T3, T7, or SP6 mMESSAGE mMACHINE transcription kit (Ambion, Austin, TX, USA). Depending on the channel type, defolliculated oocytes were injected with 10-50 nL of cRNA at a concentration of 1 ng/nL using a micro-injector (Nanoject II; Drummond Scientific Company, USA). Following the cRNA injection and 1-5 days of incubation at 16°C in ND96 buffer, electrophysiological experiments were conducted.

### 2.6 Subcloning of B2R in high expression vector pGEM-HE

For heterologous expression of bradykinin 2 receptor (B2R in pcDNA3.1+) in *Xenopus laevis* oocytes, the cDNA fragment was subcloned into a high expression vector pGEM-HE which contains 3’ and 5’ untranslated regions of a *Xenopus* globin gene (11). The B2R insert was generated by a double analytic digest with XbaI and BamHI, two restriction sites that flanked the insert. Sticky ends in the vector pGEM-HE were made by cleaving the restriction sites with XbaI and BamHI. Following, the B2R insert was ligated into the linearized pGEM-HE construct with T4 ligase (Thermo Fisher Scientific, USA). After ligation, competent *E. coli* JM109 cells (Promega, USA) were transformed to express the corresponding cDNA. Next, the cDNA was linearized with XbaI and cRNA was transcribed with a T7 mMESSAGEmMACHINE transcription kit (Ambion, Austin, TX, USA).

### 2.7 Electrophysiological recordings with a two-electrode voltage-clamp

Electrophysiological experiments were performed using a two-electrode voltage-clamp (TEVC) GeneClamp 500 amplifier (Molecular Devices, Downingtown, Pennsylvania, USA) controlled by a pClamp data acquisition system (Axon Instruments, Union City, California, USA) connected to a computer equipped with a Windows XP operating system (Microsoft^®^, USA) and Camplex9 software (Axon Instruments^®^, USA), enabling data acquisition and storage. Whole-cell currents from oocytes were recorded at room temperature (18-22°C) after 1-5 days of cRNA injection. Voltage and current electrodes were pulled from borosilicate glass capillaries by a microelectrode puller, PUL-1 (World Precision Instruments, Sarasota, FL, USA). Both capillaries were filled with 3 M KCl by using a MicroFil needle. With the micromanipulator, the electrodes were accurately positioned and submerged into a bath filled with the ND96 solution ready to impale the oocyte. The resistance of the electrodes was maintained between 0.5 MΩ and 1.5 MΩ. A membrane test was first performed to adjust measurement parameters as a function of the quality of the membrane. For electrophysiological recordings, a number of protocols were applied from a holding potential of -90 mV, -70 mV or -20 mV (depending on the experiment).

Lyophilized crude venom extract of *A. zaruma*, was resuspended in ND96 solution or HK solution (depending on the experiment) to make a stock concentration of 10 µg/µL. For all experiments, the amount of 40 µg crude venom extract of *A. zaruma*, was applied by pipetting directly on a 200 µL bath, under no flow, and with a final concentration of 0.2 µg/µL. The venom was incubated for ~60 s in the recording chamber with the oocyte.

#### 2.7.1 Electrophysiological recordings of voltage-gated sodium and potassium channels

For voltage-gated sodium and potassium channel protocols, the elicited currents were sampled at 20 kHz for Nav1.x, 10 kHz for Kv1.x, hERG and *Shaker*-IR. *Via* a four-pole low-pass Bessel filter, the currents were filtered at 2 kHz for Nav1.x, 500 MHz for Kv1.x, and 1 kHz for hERG and *Shaker*-IR. Leak subtraction was performed using a -P/4 protocol.

Nav1.x traces were evoked by 100-ms depolarizations to the voltage corresponding to maximal Na+ current in control conditions (V_max_). The current-voltage relationships were investigated by 50-ms step depolarizations between −90 and +40 mV, using 5 mV increments. For the inactivation, a two-step protocol was employed, with a 100-ms conditioning pulse ranging from -90 mV to 0 mV with a 5 mV step. This was immediately followed by a test pulse to 0 mV. Next, Kv1.x and *Shaker*-IR currents traces were evoked by 500-ms depolarization pulses to 0 mV followed by 500-ms repolarization pulses to -50 mV. hERG1 current traces were evoked by the application of +40 mV pre-pulses for 200-ms, immediately followed by a step of -120 mV for 200-mV.

#### 2.7.2 Electrophysiological recordings of ligand-gated ion channels and receptors

Oocytes were placed in a 200 µL recording chamber with ND96. The agonist, antagonist and venom application/washout are controlled by a perfusion system that rapidly switches the perfusion of the syringe reservoirs and with a gravity flow at ~1 mL/min. A manifold that connects multiple syringe reservoirs was used.

##### 2.7.2.1 GIRK1, GIRK2 and IRK1 measurements

For GIRK1, GIRK2 and IRK1 measurements, currents were induced by exchanging low potassium (ND96) solution with high-potassium solution (HK; 96 mM KCl, 2 mM NaCl, 1 mM MgCl_2_, 1.8 mM CaCl_2_, 5 mM HEPES with a final pH of 7.5) while the oocytes were voltage-clamped at -90 mV. The elicited currents were sampled at 100 Hz and *via* a four-pole low-pass Bessel filter filtered at 20 Hz. The increase in current represents a basal K_+_ current that is following receptor-independent GIRK/IRK channel activation (I_K_, _basal_).

##### 2.7.2.2 G-protein coupled receptors: GIRK1 and GIRK2 coupled with H2-4R, MRGPRX2 or CB2

The receptors H2-4R, MRGPRX2 and CB2 were coupled to the inward rectifier potassium channels (GIRK1 and GIRK2) *via* a Gi/o cascade in order to observe the agonist produced reversible receptor-dependent K^+^ current in a controlled and stable manner (I_K_, _agonist_). Currents were measured in HK solution using a protocol of -90 mV during 400 s, sampled at 100 Hz and filtered at 20 Hz. During the measurement 2 pulses of the agonist (H2-4R: 1 µM histamine; MRGPRX2: 10 µM compound 48/80 and CB2: 1 µM WIN55, 212-2) with ~ 30 sec of interval between each pulse were applied as a control for each individual experiment. This was followed by a cell washout of ~ 3 min and a venom application. The venom was applied until a stable activated state of the channel was reached (when the agonist activity was visible) or at least 1 min when no significant current increase was visible. Then, the venom was washed out by HK followed by a wash-out with ND96. After venom application, the agonist was applied again to test reversibility.

##### 2.7.2.3 nAChR and ASIC1a measurements

For muscle-type α1β1δϵ and neuronal-type α7 nAChR, the oocytes were clamped at a holding potential of -70 mV, sampled at 100 Hz and filtered at 20 Hz. At least three control responses of 100 µM acetylcholine (ACh) were assessed prior to the application of the venom. After 30 s of washing, the venom was applied for ~60 s at 1 mL/min, immediately followed by a pulse of 100 µM ACh. Also, for ASIC1a channel, a protocol with a holding potential of -70 mV, a sampling rate at 100 Hz and a filter frequency of 20 Hz was used. Currents were evoked by exchanging ND96 solution pH of 7.5 with ND96 solution pH of 4.5. At least three control responses of pH 4.5 ND96 solution were assessed prior to the application of the venom. After 30 s of washing, the venom was applied for ~60 s at 1 mL/min, immediately followed by a pulse of pH 4.5 ND96 solution.

##### 2.7.2.4 TRPV1 measurements

For TRPV1, currents were monitored in continuously perfusing ND96 solution using a protocol of -90 mV during 400-s, sampled at 100 Hz and filtered at 20 Hz. Capsaicin (10 µM) was used as agonist for TRPV1.

##### 2.7.2.5 H1R and B2R measurements

Finally, H1R and B2R currents were sampled at 1000 Hz, filtered at 20 Hz and measured using a 2-s voltage ramp protocol applied from -120 to +70 mV from a holding potential of -20 mV during perfusion. 1 µM histamine and 1 µM bradykinin were used as an agonist for H1R and B2R respectively.

### 2.8 Quadrupole time-of-flight mass spectrometry (Q-TOF-MS)

*A. zaruma* venom extract was analyzed by a bottom-up proteomic approach using positive electrospray ionization (ESI) performed on quadrupole time-of-flight (Q-TOF) mass spectrometer (Bruker Impact II, Bruker Daltonics, Germany). Samples were prepared as follows: lyophilized crude venom extract of *A. zaruma*, was resuspended in water for a total stock concentration of 10 µg/µL. 100 µL of the stock solution was dissolved in 200 µL of 100% acetonitrile and cleared by centrifugation at 10 rpm for 5 min. 300 µL of supernatant was dissolved in 700 µL ddH_2_0 to get a final concentration of 1 mg/mL *A. zaruma* venom extract (1/10 dilution). Different concentrations for histamine samples were prepared according to the same protocol: 0.1 µM, 1 µM, 10 µM and 100 µM. The Q-TOF system was operated in positive mode using Bruker TargetScreener HR 4.0. This program includes hardware, column and methods with a total analysis time including chromatographic separation and accurate mass detection of 20 min (12). During data acquisition, both MS and MS/MS full scan mode datasets were obtained. The MS raw data were obtained in format from Bruker Compass Data Analysis Viewer version 5.2 (Bruker Daltonics, Germany).

### 2.9 Data analysis

All electrophysiological data were obtained using pClamp Clampex 10.4 (Axon Instruments, San Jose, CA, USA) and analyzed using pClamp Clampfit 10.4 (Axon Instruments, San Jose, CA, USA). Concentration-response curves were fitted to a four-parameter Hill equation with a variable Hill coefficient. Data are presented as means ± standard deviation (SD) of n ≥ 3 independent experiments. For all voltage-gated sodium channel experiments: Na+ conductance (g_Na_) was calculated using Ohm’s law: g_Na_ = I_Na_/(V −V_rev_), where I_Na_ represents the Na+ current peak amplitude at a given test potential V, and V_rev_ is the reversal potential. The values of g_Na_ were plotted as a function of voltage and fitted using the Boltzmann function: g_Na_/g_ma*x*_= [1+ exp(V_g_−V)/k)]^−1^ , where g_max_ represents maximal g_Na_, V_g_ is the voltage corresponding to half-maximal conductance and k is the slope factor (13).

## 3 Results

### 3.1 *Automeris zaruma* is covered with large hollow spines that serve as a storage room for the venomous cocktail

**Figure 1:**
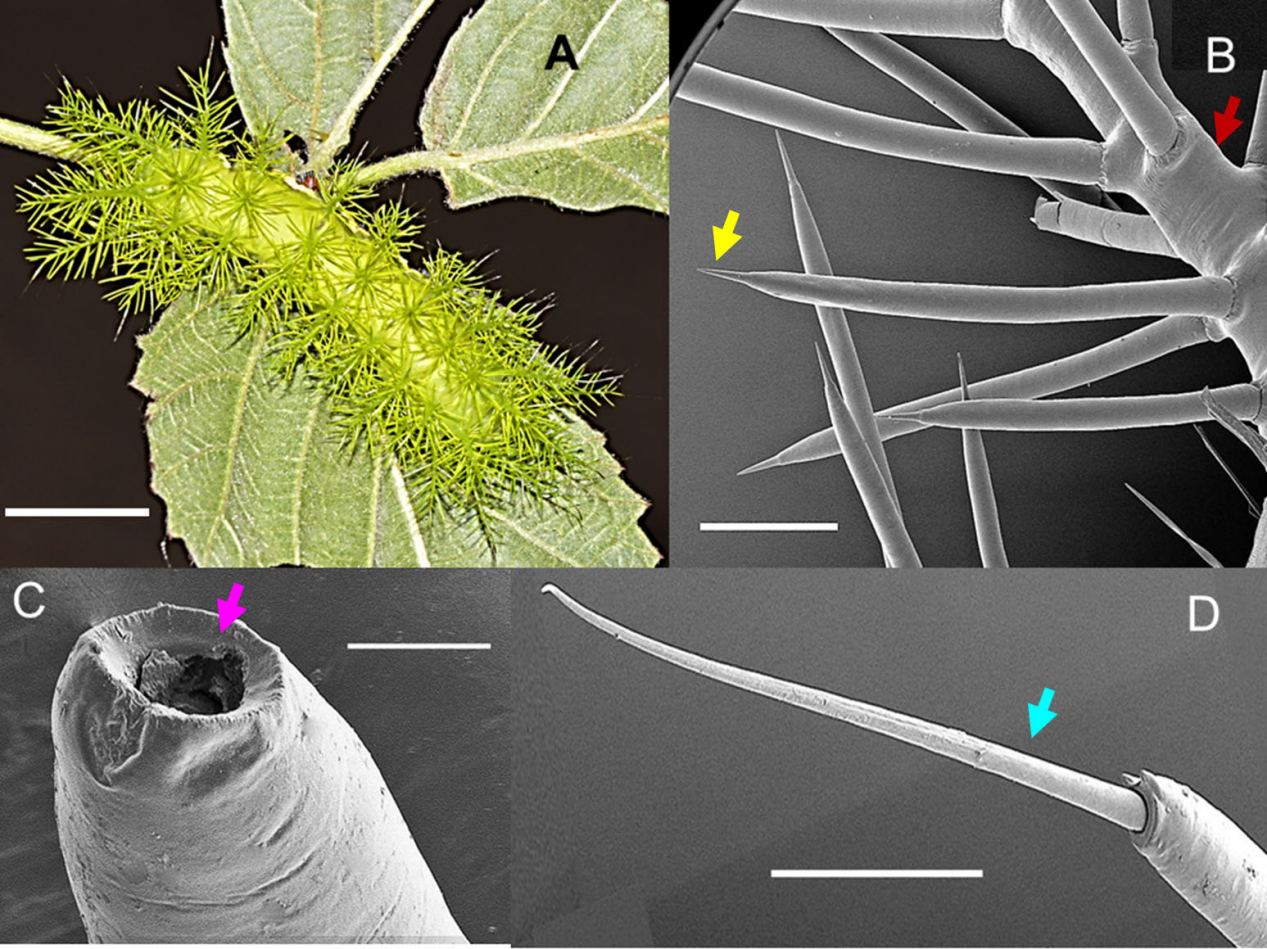
The caterpillar of the moth *Automeris zaruma*. **(A)** Mature larva covered with numerous dorsal and subdorsal spines (bar – 1 cm). Scanning electron microscopy of **(B)** stinging spines with a sharp-pointed tip (yellow) branching off a long skin tubercle (scolus, red) (bar – 600 µm). **(C)** The tip of the hollow spine (bar - 40 µm, pink). **(D)** setae-bearing spines (blue): smaller spines with a long and flexible tip that originates from the scolus (bar- 200 µm).

Mature larvae of *A. zaruma* are covered with numerous large venomous spines at their lateral, dorsal and subdorsal site. Both, the dorsal and subdorsal spines are the first to come in contact with the skin of the victim and are thus the spines of interest. They are morphologically identical to the lateral spines. Scanning electron microscopy showed these spines which measure up to ~0.5 cm in length and have a sharp-pointed tip branching off a long skin tubercle (scolus, red arrow) (**Figure 1A**). This tip (yellow arrow) helps the envenomation by easily breaking off and releasing its venomous content from the hollow spine (pink arrow) as shown in **Figures 1B, C**. Strikingly, among the venomous spines, also smaller ones with a long and flexible tip (setae-bearing spines, blue arrow) were observed **(** **Figure 1D**). The same setae-bearing spines were also seen and described for *A. io* (10). Contact with the spines results in an almost immediate mild to severe pain (depending on the amount of venom) accompanied by local wheals, papules and welts persisting for several hours, days and sometimes weeks after the sting.

### 3.2 Histamine Receptors are key targets in the envenomation by *Automeris zaruma*

Pain, inflammation and itch are three key events in the pathogenesis of *A. zaruma* envenomation. To understand how these events are trigged by the mechanism of action, we investigated the involvement of one or more molecular target(s) in the pathway. Since, histamine is a well-known mediator of itch, swelling, urticaria, redness and burning pain we hypothesize that histamine through its action *via* the four histamine receptors may play an important role in the envenomation. Therefore, we performed functional studies on the histamine receptors using the two-electrode voltage clamp technique. In our bio-assay, 1 µM histamine and 0.2 µg/µL venom extract are applied to the four types of histamine receptors: H1R, H2R, H3R and H4R for real-time monitoring the activity.

**Figure 2:**
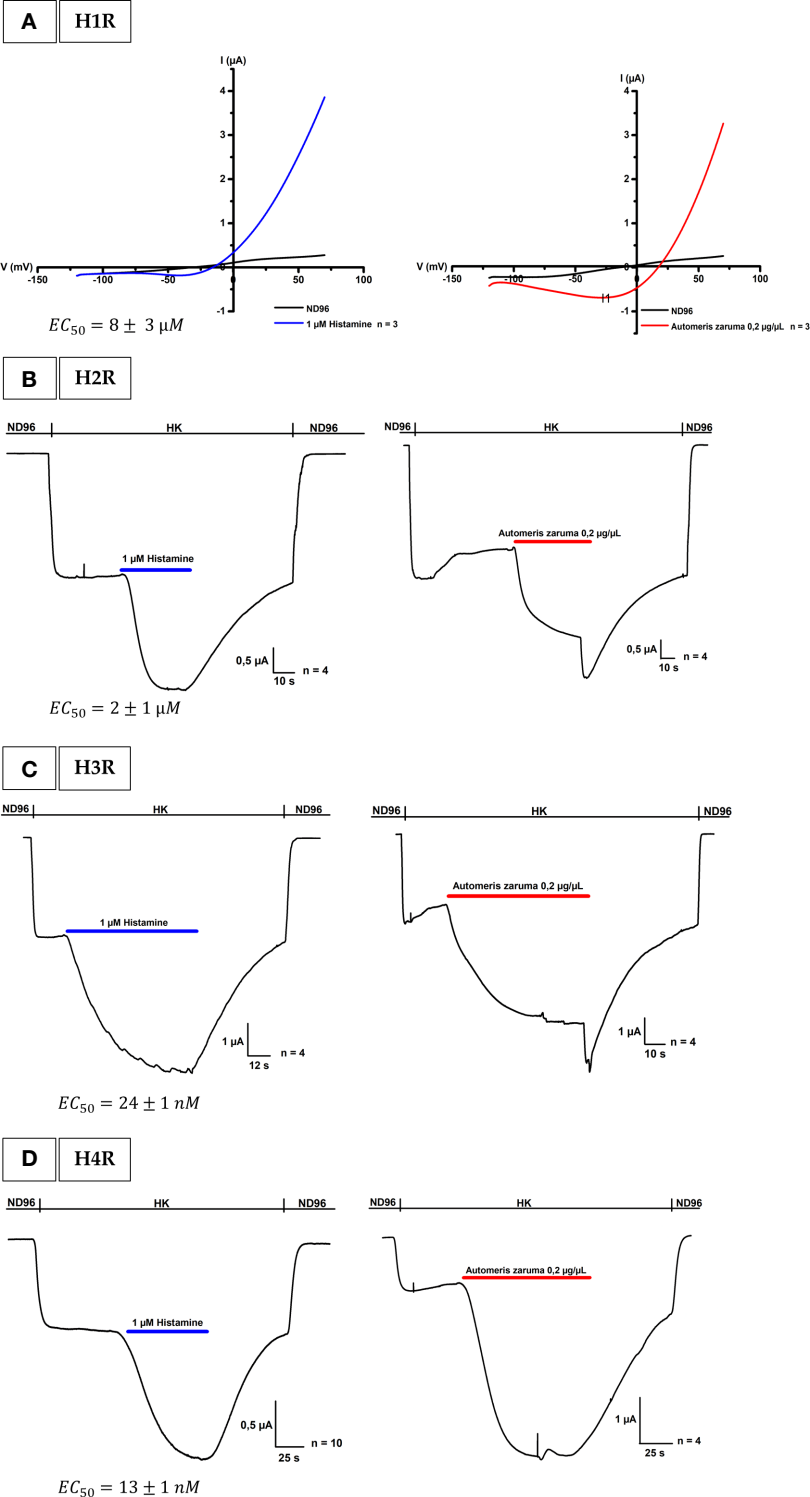
Activation of histamine receptors by *A. zaruma* venom extract in *Xenopus* oocytes. **(A)** A representative current trace of H1R. A change in conductance is observed upon application of 1 µM histamine (blue, left) and 0.2 µg/µL venom extract (red, right). **(B)** A representative current trace of H2R shows a current increase upon application of 1 μM histamine (blue, left) and in the presence of *A. zaruma* venom extract (red, right). **(C)** A representative current trace of H3R. Current enhancement is observed upon application of 1 μM histamine (blue, left) and in the presence of *A. zaruma* venom extract (red, right). **(D)** validation trace experiments of H4R, currents were induced upon application of 1 μM histamine (blue, left) and in the presence of *A. zaruma* venom extract (red, middle).

For the H1R, it is well known that it is activated by histamine through a Gαq/11 signaling (14). Using a ramp protocol, we found that 1 µM histamine in ND96 evoked a change in conductance in oocytes expressing the H1R. This change in conductance was reversible after a wash-out period with ND96 (**Figure 2A**, left). After having established the activation of the H1R, we constructed an activation response curve in order to validate the bioassay and to identify the desired concentration of histamine administration during the recordings. In this experiment oocytes were exposed to an increasing histamine concentration. The evoked currents were normalized against the saturated histamine concentration. **Supplementary Figure S1**, represents the resulting concentration-response curve for H1R in blue where the percentage of activation was plotted against the logarithm of the applied concentrations. An EC_50_ of 8 ± 3 µM was obtained which is in line with the value found in literature (EC_50_ = 24 µM) (15). Next, we investigated the effect of the venom on H1R. In **Figure 2A** on the right, it is clearly visible that an application of 0.2 µg/µL venom is able to change the conductance and thus activate the H1R. Not only an activation was visible, the outward current was also approximately the same in amplitude as the outward current evoked by 1 µM histamine as shown in **Table 1**.

**Table 1:** % Activation by histamine and *A. zaruma* venom extract at H1R, H2R, H3R and H4R.

	H1R Activation (%)	H2R Activation (%)	H3R Activation (%)	H4R Activation (%)
1 µM Histamine	93 ± 1	55 ± 11	109 ± 11	199 ± 36
0.2 µg/µL *Automeris zaruma* venom extract	92 ± 2	54 ± 10	114 ± 12	152 ± 16

Histamine and *A. zaruma* venom extract were screened at a concentration of 1 µM and 0.2 µg/µL respectively. The data are represented as the mean ± S.E.M. The experiments were repeated at least three times (n ≥ 3).

For the H2R, H3R and H4R experiments, *Xenopus* oocytes were co-injected with cRNA encoding the human H2R, H3R, or H4R and the GIRK1 and GIRK2 subunits. This created a robust and quantifiable signal as shown in **Figures 2B–D** on the left. Through this coupling *via* the Gi/o cascade, we were able to observe the agonist-produced receptor-dependent K^+^ current. More specific, when we exchanged the low potassium ND96 solution with a high potassium solution HK, we were able to observe the basal K^+^ currents (receptor-independent, I_K_, _basal_). The activation was mediated by the addition of 1 µM histamine in HK. The agonist binding to the H2-4 receptors promotes the activation of the G-proteins that interact with the effector proteins GIRK1 and GIRK2. This produces a visible increase in K^+^ current (receptor-dependent, I_K_, _agonist_). The current was reversible after a wash-out period with ND96. In the same way as H1R, also for the H2R, H3R and H4R a doses-response curve was established and represented in **Supplementary Figure S1**. For H2R colored in orange, a maximum saturated current activation was observed at a concentration of about 100 µM and the EC_50_ value was estimated to be 2 ± 1 µM which corroborates with the value reported in the literature (EC_50_ = 10 µM) (16). H3R colored in green reached a half maximum response at a concentration of 24 ± 1 nM, again perfect in agreement with the EC_50_ found in the literature (EC_50_ = 55 ± 7.89 nM) (17). The obtained EC_50_ value of 13 ± 1 nM for H4R (pink) was also comparable according to what has been described previously (EC_50_ = 13 ± 0.2 nM) (18). Important to note, a difference in potency was clearly visible in the dose-response curve. Thus, we found that histamine has a much higher affinity for H3R and H4R. An observation that once again confirms the literature. Next, we tested the effect of the venom extract on oocytes co-injected with H2R, H3R, or H4R and GIRK1 and GIRK2 cRNA. We show that the venom extract from *A. zaruma* can activate the H2-4 receptors (**Figures 2B–D** on the right). More specifically, the first current increase represents the K^+^ currents (I_k, basal_) that were evoked by exchanging solutions from ND96 to HK. The second current enhancement was evoked by the application of 0.2 µg/µL venom extract of *A. zaruma* (I_k, agonist_) and was reversible following a washout with ND96. Strikingly, the activation by the venom was again comparable to the activation by 1 µM histamine, as indicated in **Table 1**.

**Figure 3:**
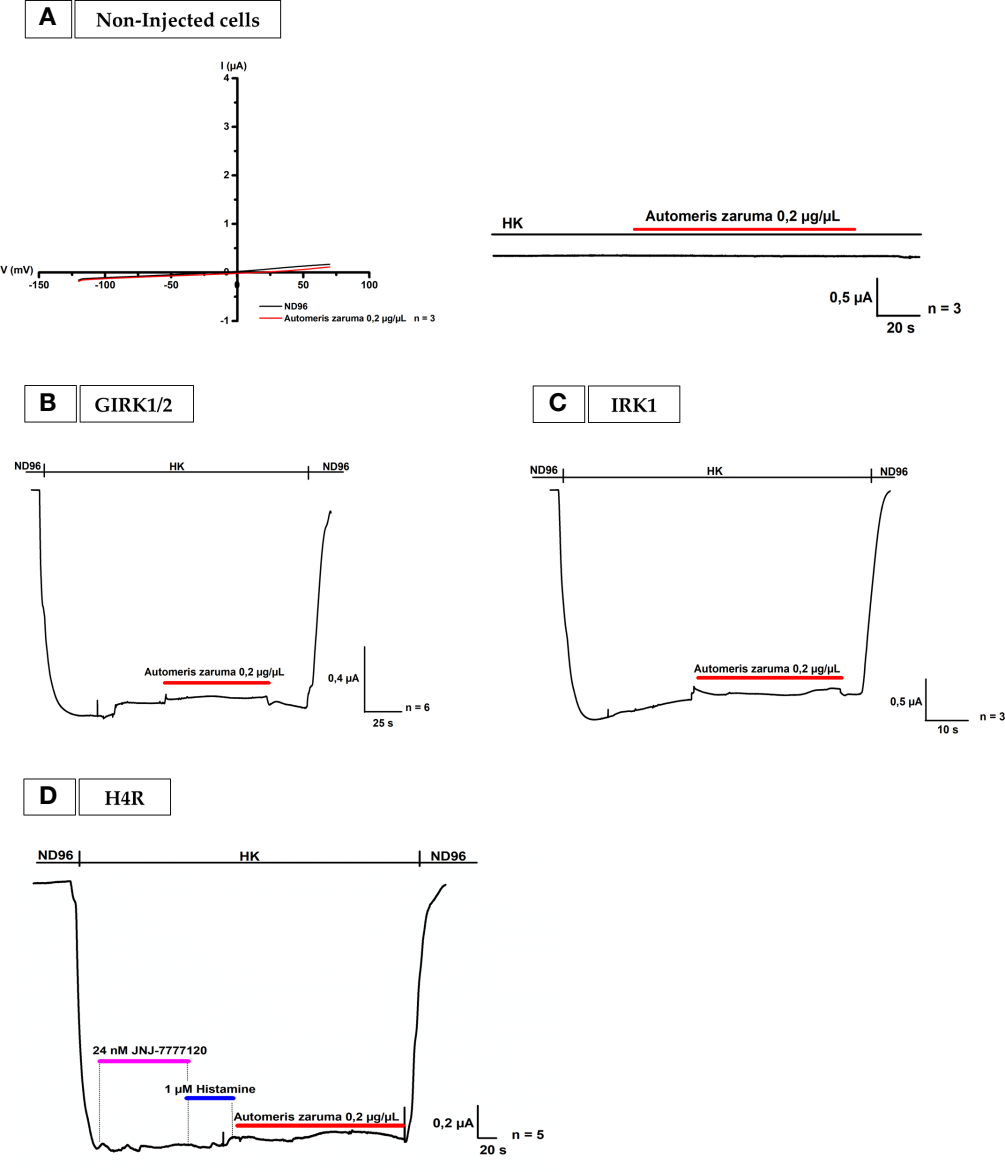
Effect by *A. zaruma* venom extract on non-injected cells, GIRK1 and GIRK2, IRK1 injected cells and in the presence of a selective H4R antagonist. **(A)** Two validation curves represent no appreciable current enhancement by *A. zaruma* venom extract (red) in non-injected *Xenopus laevis* oocytes. **(B)** A validation current trace show that no significant current was induced by *A. zaruma* extract (red) in *Xenopus laevis* oocytes injected with GIRK1 and GIRK2. **(C)** A validation curve visualizes no current increase by *A. zaruma* venom extract on oocytes injected with IRK1. **(D)** The current enhancement by histamine (blue) and 0.2 µg/µL *A. zaruma* venom extract (red) disappeared in the presence of JNJ 7777120 antagonist (pink). All experiments were repeated at least three times (n ≥ 3).

To confirm these observations, three control experiments as shown in **Figures 3A–C** were performed. Following the pathway in our bio-assay, we first tested the venom on non-injected oocytes with a ramp protocol from -120 to +70 mV from a holding potential of -20 mV in ND96 and with a voltage protocol at -90 mV in HK. In **Figure 3A** no change in membrane current is observed. Also, oocytes injected with only GIRK1 and GIRK2 did not yield any change in membrane current (**Figure 3B**). Both experiments support the notion that the venom does not act on non-injected cells and that it is not a direct activator of the GIRK1 and GIRK2 channels. In the next control experiment, the venom effect was tested on Inward-Rectifier potassium channel (IRK) (**Figure 3C**). This channel differs from GIRK channels mainly by the fact that it does not interact with G-proteins. It was observed that the venom extract did not cause a significant effect on IRK channel.

To conclude, to the best of our knowledge, we report here for the first-time functional expression of the four histamine receptors in *Xenopus* oocytes and we pinpoint H1R, H2R, H3R and H4R as important molecular targets in the envenomation by *A. zaruma*. Moreover, we observed a similar effect as 1 µM histamine, which might indicate a similar amount of concentration of histamine in the venom present

### 3.3 A potent histamine 4 receptor antagonist, JNJ 7777120, completely blocks the effect of the *Automeris zaruma* venom extract on oocytes co-injected with H4R and GIRK1/2

In the last validation experiment, the effect of the venom is tested on oocytes co-injected with H4R and GIRK1/2 in the presence of an antagonist (**Figure 3D**). In short, all H4Rs in the same cell were blocked by 24 nM JNJ 7777120. JNJ 7777120 is a selective H4R antagonist with an IC_50_ of 5 ± 0 nM (18). To prove this, 1 µM histamine in HK was applied. Because of the blockage by the H4R antagonist, no current increase could be observed. When the venom was added, the second current enhancement evoked by the application of 0.2 µg/µL venom extract also disappeared. Thus, these results support the concept that a selective blocker of H4R is able to successful block the effect of the venom of *A. zaruma* on H4R.

### 3.4 The selectivity of *Automeris zaruma*venom extract on different ion channels

To determine the selectivity of the venom for the four histamine receptors, we continued to screen broadly for other ligand-gated and voltage-gated ion channels and receptors.

#### 3.4.1 The effect of *A. zaruma* venom extract on different voltage-gated ion channels

We started with the screening of *A. zaruma* venom activities against a panel of five voltage-gated sodium channel isoforms: Na_v_1.2, Na_v_1.3, Na_v_1.4, Na_v_1.6 and Na_v_1.8. We constructed the steady-state activation and inactivation curves for Na_v_1.2, Na_v_1.4, Na_v_1.6 and Na_v_1.8 as illustrated in **Supplementary Figure S2**. In the presence of 0.2 µg/µL *A. zaruma* venom extract, no significant modulation of gating kinetics nor a change in the reversal potential was observed for the tested sodium channels. This indicates that the ion selectivity of Na_v_ is not altered by the presence of 0.2 µg/µL *A. zaruma* extract.

Next, *A. zaruma* venom extract was tested for its activity against five voltage-gated potassium (K_V_) channels: K_v_1.1-1.3, *Shaker* IR, hERG1 as shown in **Supplementary Figure S3**. At a concentration of 0.2 µg/µL, *A. zaruma* venom failed to exert any activity (inhibition or activation) against the tested K_V_ channels.

#### 3.4.2 The effect of *A. zaruma* venom extract on different ligand-gated ion channels and receptors

**Figure 4:**
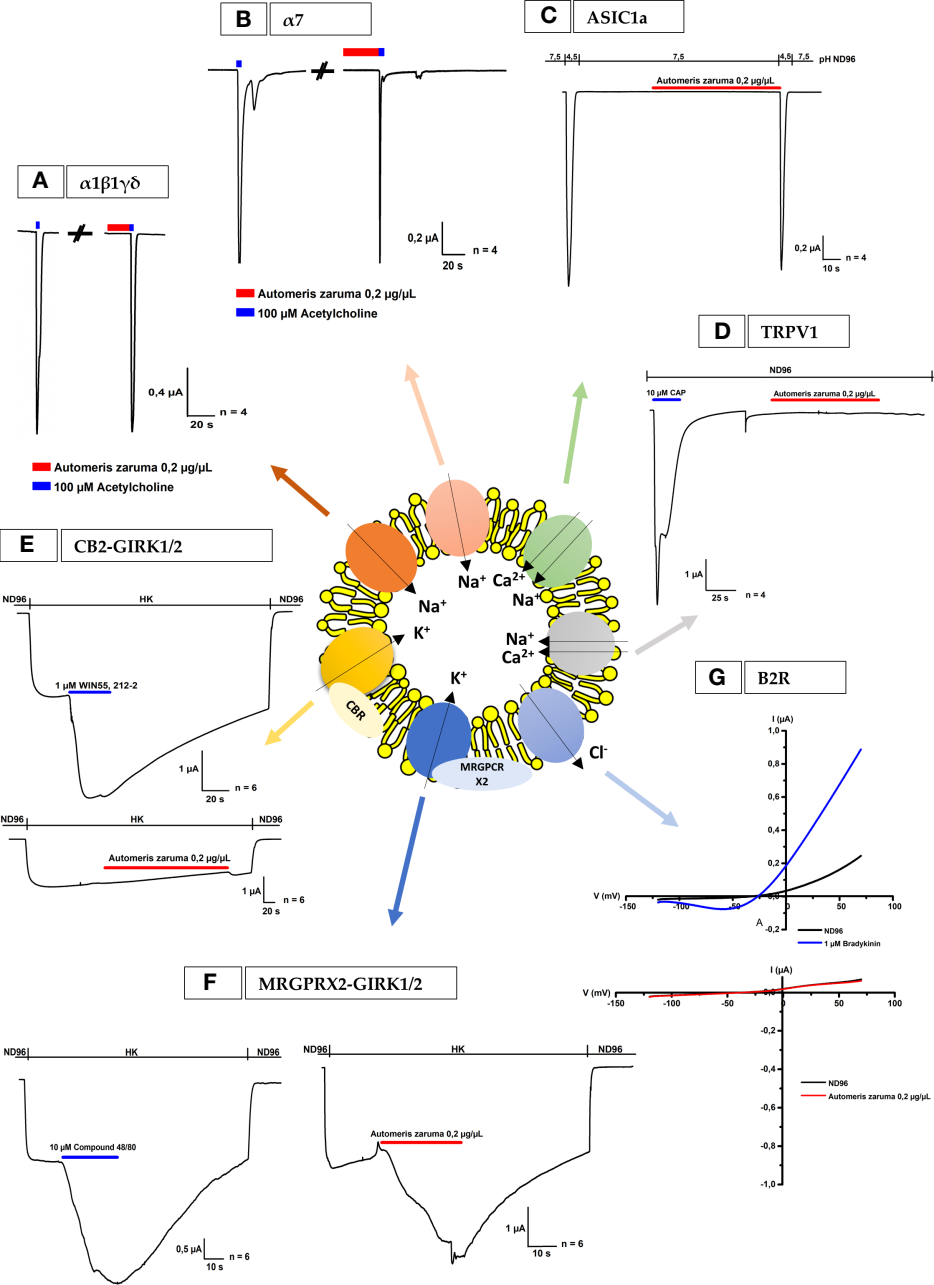
Electrophysiological activities of *A. zaruma* on nAChR, ASIC1a, TRPV1, B2R and oocytes co-expressing CB2 or MRGPRX2 and GIRK1 and GIRK2. **(A, B)** Electrophysiological profile of muscle-type α1β1γδ nAChR and neuronal-type of α7 nAChR. The nAChRs were gated by 100 µM ACh (blue) at 1 mL/min. The first and the second peak amplitude represent the absence and presence of 0.2 µg/µL of *A. zaruma* venom. **(C)** Whole-cell ASIC1a current traces in control pH 4.5 and in the presence of *A. zaruma* venom extract in pH 7.5 and pH 4.5. **(D)** Lack of effect of 0.2 µg/µL *A. zaruma* venom extract on TRPV1 channels. The first peak amplitude represents the presence of the 10 µM Capsaicin agonist. **(E)** A representative current trace of CB2-GIRK1/2. Current enhancement is observed upon application of 1 μM WIN55,212-2 (blue) but no significant effect was visible in the presence of 0.2 µg/µL *A. zaruma* venom extract (red). **(F)** Validation trace experiments of MRGPRX2-GIRK1/2, currents were induced upon application of 10 μM compound 48/80 (blue, left) and in the presence of 0.2 µg/µL *A. zaruma* venom extract (red). **(G)** A representative trace of 1 µM bradykinin evoked a change of conductance (blue) and no significant trace was evoked by 0.2 µg/µL *A. zaruma* extract on B2R. All experiments were repeated at least three times (n ≥ 3).

Following the voltage-gated ion channels, several ligand-gated ion channels and receptors were tested and presented in **Figure 4**. First, the pharmacological profile and effect on the ACh-evoked current were investigated for *A. zaruma* venom extract on *Xenopus* oocytes expressing the muscle-type of α1β1γδ nAChR and the neuronal-type of α7 nAChR (**Figures 4A, B**). Interestingly, no significant change in current amplitude was observed after the application of 0.2 µg/µL *A. zaruma* venom extract. Next, the effect of *A. zaruma* venom extract (0.2 µg/µL) was tested on ASIC1a channel expressed in *Xenopus* oocytes as shown in **Figure 4C**. At a pH of 7.5 and pH 4.5, again no significant modulation in current could be observed upon application of 0.2 µg/µL venom extract. In the same figure, also the results for the measurements of TRPV1 channels are presented (**Figure 4D**). Here, it is clearly visible that 0.2 µg/µL *A. zaruma* venom extract was not able to show any agonist or antagonist activity on TRPV1 channels.

Since the H2R, H3R and H4R are G-protein coupled receptors (GPCRs), we wondered if other GPCRs also would be susceptible to the venom of *A. zaruma*. Therefore, we tested the cannabinoid receptor (CB2) and the Mas-Related G protein-coupled receptor X2 (MRGPRX2). These receptors were co-expressed with GIRK1 and GIRK2 channels and the representative current traces are visualized in **Figures 4E, F**, respectively. Here, the first graph represents the activation of the channel by the agonist. More concrete, the first current increase represents the K^+^ currents (I_k_, _basal_) that were evoked by exchanging the ND96 buffer with HK solution. The second current enhancement was evoked by the application of the agonist: 1 µM WIN55,212-2 for CB2 and 10 µM compound 48/80 for MRGPRX2. In the second graph, the effect of *A. zaruma* venom extract was shown. Once again, the first current increase represents the K^+^ currents (I_k_, _basal_) that were evoked by increasing the extracellular K^+^ concentration. For CB2, the venom extract of *A. zaruma* (0.2 µg/µL) failed to exert any activity (**Figure 4E**). Very interesting, for MRGPRX2, an agonist activity of the venom was visible *via* the second current enhancement evoked by 0.2 µg/µL *A. zaruma* venom extract (**Figure 4F**). This was reversible following a washing out with HK and ND96. Strikingly, 1 µM histamine doesn’t have any effect on MRGPRX2 (**Supplementary Figure S4**).

Finally, we tested the effect of *A. zaruma* venom on *Xenopus* oocytes injected with cRNA encoding the bradykinin 2 receptor (B2R) (**Figure 4G**). Oocytes were subjected to a 2-s voltage ramp protocol from -120 to +70 mV from a holding potential of -20 mV. A change in conductance (large outward current and a small inward current) was observed upon exposure to 1 µM bradykinin and was reversible on removal *via* washing out with ND96. Next, the oocytes were exposed to 0.2 µg/µL *A. zaruma* venom extract. No significant activity was observed upon application of 0.2 µg/µL *A. zaruma* venom extract.

### 3.5 The discovery of histamine in the venom extract of *Automeris zaruma via* Q-TOF-MS

To better understand the molecular basis for the observed pharmacological activity, we prepared a venom extract of *A. zaruma* for analysis on the ESI-Q-TOF mass spectrometer. This revealed the presence of histamine in the venom (**Supplementary Figure S5**). The first LC-MS chromatogram (**Supplementary Figure S5A**) visualizes the separation of the *A. zaruma* venom extract where the peak intensity is plotted against the retention time. *Via* the Bruker Compass Data Analysis (Version 5.2) software tool, the molecular formula of histamine (C_5_H_9_N_3_) was predicted with a retention time of 1.18 min. From this, the extracted ion chromatogram of histamine in *A. zaruma* extract and 10 µM histamine was obtained and shown in **Supplementary Figures S5B and S5C**. The MS/MS fragmentation spectrum of the molecular ion is presented with the most abundant peak at *m/z*_measured_ = 112.0868 and an intensity of about 4.2 x 10^5^. This is in line with the specific mass-to-charge ratio m/z 112.1 described in the literature (19).

In an attempt to quantify the amount of histamine in the venom extract, the extracted ion chromatogram of histamine in *A. zaruma* venom extract was compared to the chromatogram of the different histamine concentrations (0.1 – 100 µM). Visually, it was clear that the concentration of histamine in the venom extract with a peak area of 7031600 was situated somewhere between 10 µM (peak area: 999530.75) and 100 µM (peak area: 15473564) histamine. To determine the concentration of histamine in the venom extract, the peak areas were plotted versus the concentration in a calibration curve (**Supplementary Figure S6**) with formula (1).


(1) y=0.00000621803x + 3.7849148411 

This resulted in a corresponding concentration of 47,5076 µM histamine found in the venom of *A. zaruma* extract with a stock concentration of 10 µg/µL. For all electrophysiological measurements, the amount of 40 µg crude venom extract of *A. zaruma*, was applied by pipetting directly on the 200 µL bath with a final concentration of about 0.2 µg/µL. This means that a concentration of ~1 µM histamine is present in 0.2 µg/µL *A. zaruma* venom extract during the electro-physiological measurements.

## 4 Discussion

In this modern world of medicine, caterpillar envenomation is still a locked treasure trove. Over many years, some researchers took the path to investigate their venom to characterize the venom composition and to get a notion of the pathogenesis. Undeniable, it is not easy to link the provoked symptoms in patients with the testimony of relevant bioactive molecules inside the venom. Although, a better understanding of the mechanism of action of its composing venom components is important to fulfill the unmet therapeutic need to successfully treat caterpillar envenomation.

The present study reports for the first time an active compound in the venom and the study of the bio-activity of *Automeris zaruma* caterpillar venom. *A. zaruma* is armed with stinging spines filled with a venomous cocktail that is used as defense against predators. The needle-shaped spines are sharp enough to serve as an injection device for the venom to exert local and/or eventually systemic pathological effects. Interestingly, among the venomous spines, smaller spines were observed. These smaller spines were also seen in *A. io* (10). However, it is unlikely that they are also involved in venom application but may rather represent a sensory sensillum. Even though, these spines originate from the scolus, venom may also be released when the setae break off.

Typically, contact with the spines produce an immediate local pain lasting for hours to some days. The area of skin contact remains sensitive reacting with moderate pain when touched, and erythema and pruritic wheals appear. Keeping these symptoms and their pathogenesis in mind, we investigated the possible involvement of 13 voltage-gated and 11 ligand-gated ion channels and receptors in the pathway.

The most striking effect we observed was the activation of the histamine receptors 1, 2, 3 and 4 by the venom extract. This action could be completely blocked by a specific histamine receptor antagonist as demonstrated in the experiment with the H4R antagonist, JNJ 7777120 using oocytes co-injected with H4R and GIRK1 and GIRK2. Thus, the data support the concept that the H1R, H2R, H3R and H4R are important molecular target(s) of the venom from *A. zaruma*. More importantly, we observed a similar effect as 1 µM histamine, which might indicate a similar amount of concentration of histamine present in the venom.

In line with these results, we confirmed that the venom of *A. zaruma* contains histamine by Q-TOF-MS analysis, which is present at a concentration of 1 µM histamine in 0.2 µg/µL venom extract. These findings provide evidence of the qualitative and quantitative correlation between the symptoms of *A. zaruma* envenomation, histamine present in the venom and its activity on the four histamine receptors (H1R, H2R, H3R and H4R).

Histamine is well known to play an essential role in stimulating inflammation, redness, itching, swelling and urticaria *via* its interaction with the histamine receptors (H1R, H2R, H3R and H4R) (20). These receptors belong to the G-protein coupled receptor family which contains 7 transmembrane domains and are expressed in a large variety of cell types of the skin (21). Clinically, H1 and H4 receptors mediate pain, inflammation, urticaria, vasodilation and redness. They are involved in diseases such as atopic dermatitis, allergic asthma and other inflammatory diseases (22). The H2 receptor is predominantly responsible to induce airway mucus production and secretion of gastric acid while the H3 receptor plays an important role in inflammation and neurophysiological disorders (21). Probably, most of the effects are caused by the H1 and H4 receptors, while the H2R and H3R will have a minor role. However, further research is required to investigate this more in detail.

For many caterpillars it is known that envenomation is a complex clinical manifestation that not only involves local symptoms but also systemic symptoms (1). This is also true for other species from the same genus as *A. zaruma*. For example, for *A. io* it has been described that they cause systemic symptoms such as paresthesia, radiating pain, dizziness, diaphoresis, nausea, abdominal pain, muscle spasms, joint stiffness, or lymphadenopathy (10). To the best of our knowledge, for *A. zaruma* there is no information available regarding the systemic symptoms, but the venom composition may be similar and thus that the described symptoms may be present and follow the activation of the four histamine receptors. For example, for H1R it is know that the stimulation of H1R in the brainstem can evoke vomiting and nausea while for H2R a clinical antagonist can improve the discomfort or pain that occurs in the upper abdomen (21, 23). In some cases, H3R antagonist may help against the feeling of spinning and H4R activation may cause joint destruction (24, 25).

In contrast to the several studies on the role of histamine in the envenomation of scorpions, snakes and bees, only few studies focus on the participation of histamine in that caused by caterpillars (26–28). Shama *et al.* (1982) and Dinehart *et al.* (1987) used an enzymatic isotopic assay to quantify 80 ng histamine for the caterpillar of the gypsy moth, *Lymantria dispar*, and 2,7 µM histamine for that of the yellowtail moth, *Hylesia* spp (29, 30). For *Euproctis* caterpillars, between 0.19% to 0.48% histamine was determined in a spine extract (31, 32). Comparatively, in the venom of honey bees and yellow jacket wasps, about 0.9% and 4.6% of histamine, respectively, was found (33).

It is surprising that for two other *Automeris* species, i.e. *A. io* and *A. incisa*, no histamine or its action in histamine-mediated reactions has been identified (3, 10). As explanation of the histamine-negative results, the failure of the current antihistaminic drugs to treat the envenomation has been suggested (10).

Today, antihistaminic drugs such as bellozal and levocetirizine are widely used and are mainly blocking the H1 receptor. Since these H1 antagonists failed to attenuate all effects of the venom, it may be possible that the major envenoming symptoms are not mediated by the H1 receptor only or the selectivity window of histamine in the venom is limited towards the histamine receptors.

It is well known that histamine has a much higher affinity for the H3 and H4 receptors compared to H1 and H2 receptors (21). This can be explained by the interaction of the imidazole ring with the glutamate residue present in transmembrane 5 (21). In the present study, these differences in affinity were also confirmed by the determination of EC_50_ values ranging from high to low affinity: 13 ± 1 nM (H4R), 24 ± 1 nM (H3R), 2 ± 1 μM (H2R), 8 ± 3 µM (H1R), which suggests that a certain concentration is needed to result in 50% of the maximum response. A concentration of 1 µM histamine in the venom is shifting its selectivity and efficiency towards the H3R and H4R indicating a lower potency towards H1R and H2R. Therefore, the H3 and H4 receptors may play a more important role than the H1 and H2 receptors in the mode of action of *A. zaruma*. Consequently, for effective treatment of *A. zaruma* envenomation the present data suggest the combination of drugs at least for H1R and H4R as targets. Whether, blocking all four histamine receptors are necessary to alleviate all envenoming symptoms needs to be tested. At present, the role of H2R and H3R in mediating itch, pain and inflammation remains poorly understood (34).

Currently, several H3R and H4R antagonists have been described and some of them are being used as medicines (14, 35, 36). The H3R antagonist, Pitolisant, is approved for the clinical treatment of narcolepsy and several other antagonists are in development phase (37). However, there is a limited amount of literature on this class of drugs. Hence, more research is needed to unravel the role of H3R in pain, itch and inflammation and to see if Pitolisant could be used in these pathogeneses. Next, as described previously, for H4R, a selective antagonist, JNJ 7777120, was developed showing its vital role in reducing pruritus and inflammation. JNJ 7777120, is a great tool to understand the function of this receptor but due to the short *in vivo* half-life the clinical studies have been terminated (38). Until today, none of the developed H4R antagonists are in clinical use. However, we believe that a therapy including these drugs may not only be beneficial in treating caterpillar envenomation but also for other dermatitis-and inflammation-related diseases (39).

On the other hand, histamine receptors may not the only key players in the envenomation by *Automeris* venoms as suggested previously, such as the involvement of mediators like acetylcholine and kinins (10). Therefore, we tested the venom extract on the muscle-type of α1β1γδ nAChR, the neuronal type of α7 nAChR and the B2R. However, no significant changes in the currents were observed, indicating that there is no specific modulation of the nAChR and B2R, at least at the tested concentration present in the venom. Furthermore, Q-TOF-MS analysis confirmed the absence of acetylcholine in the venom. Additional screening on 13 voltage-gated ion channels, TRPV1 and ASIC1a also revealed no significant modulation at the tested concentration. Although, TRPV1 is important for cell depolarization, pain, stinging and itching sensation (7). These experiments confirm the selectivity of the venom for the histamine receptors.

However, further exploration of the selectivity for other GPCRs led to interesting findings. The venom extract of *A. zaruma* exerted an agonist activity at the human MRGPRX2 without affecting other GPCRs such as CB2. MRGPRX2 is a recently discovered GPCR that gained increasing scientific interest in the past few years (40). Because of its abundant expression in skin mast cells, it is placed in the forefront as receptor important for inflammatory pain and pseudo-allergic reactions (41–43). Pain is one of the key events in the pathogenesis of *A. zaruma* envenomation and in a recent publication it was shown that MRGPRB2/X2 is an important target for treating pain. In this work it was found that MRGPRB2 is a downstream effector of neuronal signaling after tissue injury (41). When MRGPRB2/X2 is activated by the neuropeptide Substance P, multiple pro-inflammatory cytokines and chemokines will be recruited *via* MRGPRB2/X2 rather by the NK-1-receptor. Taken this into account, it is possible that the venom contains a component, which activates the MRGPRX2 causing the same immunological events that eventually lead to pain. Moreover, the role of MRGPRX2 in pseudo-allergic reactions have been described (44), which are comparable with reactions caused by the activation of the histamine receptors. But in this pathway, the reactions are not mediated by histamine. This was also reconfirmed in the present experiment where histamine was not able to activate the MRGPRX2. Thus, in the venom cocktail, there is another bio-active component present that may play an important role in the signaling pathway of *A. zaruma* envenomation. Thanks to the work of Cao et al. (2021), the cryoEM structure of MRGPRX2 has been elucidated. This allowed novel insight into the signaling and binding pocket of this receptor. It was demonstrated that the MRGPRX2 binding pocket is mainly build of a negatively charged and hydrophobic sub-pocket (45, 46). This may explain why the receptor is potently activated by small cationic molecules and peptides with amphipathic properties. The fact that we see activation of MRGPRX2 by the venom of *A. zaruma* makes it interesting to speculate that the active component in the venom probably has the same characteristics and interact with the MRGPRX2 *via* ionic interactions. On the other hand, free amino acids such as arginine may be present in the venom and interact with the negatively charged binding pocket. For several venomous animals such as snakes and the honey bees, it has been described that their venom contains free amino acids (47, 48). However, more research is needed to characterize the bio-active component that activates the MRGPRX2. Transcriptomic linked with proteomic studies might fill the gaps and will provide essential information about the structure of the bio-active components. On the other hand, it is also important to further confirm the data of MRGPRX2 activation by immunological experiments. The study of calcium flux or mast cell degranulation in response to the venom may provide important information.

Regarding the role of the MRGPRX2 in pseudo-allergic reactions and pain, several researchers studied the effect of specific antagonists that block this receptor to treat these diseases. Cao et al. (2021) described C9-6 and C9, derived from Compound 2 (ZINC16991592), as novel, potent and selective MRGPRX2 antagonist with a Ki value of 58 nM and 43 nM, respectively. This compound is able to inhibit MRGPRX2 activation when stimulated by various endogenous peptides (45). Also, natural products are described to inhibit MRGPRX2 responses *in vivo* and *in vitro* and may provide a safer alternative to treat the conditions. One of them is Osthole (7-methoxy-8-isopentenoxycoumarin), a natural coumarin present in the fruits of the Cusson plant and shows a great potential in treating pseudo-allergic reactions (49). However, more research is still needed to improve the selectivity and potency towards the receptor, to optimize the bio-availability and to perform toxicity studies (50).

Until today, no specific antagonists for MRGPRX2 are in clinical use. We hypothesize that the co-administration of a histamine receptor and a MRGPRX2 antagonist may have additional benefits for the treatment of *A. zaruma* envenomation.

To conclude, we report a detailed pharmacological characterization of the venom from caterpillar *A. zaruma*. Functional studies on histamine receptors (H1R, H2R, H3R and H4R) and quantitative studies with Q-TOF-MS revealed the presence of the 1 µM histamine in venom (0.2 µg/µL). Further selectivity studies illustrate that, at least at the tested concentrations, for the first time a specific or potent modulator has been found for the histamine receptors in the venom which does not exclude the presence of other active ligands. As presented here with the modulation of MRGPRX2 by the venom, also other bio-active components and pathways are involved in the envenomation by *A. zaruma*. More research on MRGPRX2 might reveal important information. Although other active substances have not yet been identified and the site of interaction with MRGPRX2 remains to be elucidated, the present data provide evidence for altering the recommended treatment option for individuals exposed to *A. zaruma* venom. Our results namely support that the ideal treatment for this envenomation would be one that effectively inactivates the actions of histamine receptors. The hurdle of the scarce efficacy of the current antihistaminic drugs can be overcome by adding selective blockers for at least the H4R and in a minor role for H2R and H3R in the clinically used medication. Perhaps the co-addition of a MRGPRX2 antagonist may have additional benefits than histamine receptor blockers alone. Such an approach may be used for other caterpillar envenomations and may represent a significant improvement for the well-being of the patient.

## Data availability statement

The raw data supporting the conclusions of this article will be made available by the authors, without undue reservation.

## Ethics statement

The animal study was reviewed and approved by KU Leuven Ethical Committee for animal research (Project No. P186/2019).

## Author contributions

Conceptualization, AS and JT; methodology, AS, JT, and SP; formal analysis, AS; investigation, AS; resources, AS and JT; data curation, AS, JT, and SP; writing—original draft preparation, AS; writing—review and editing, AS, JT, SP, and DM; visualization, AS; providing caterpillar spines, DM; supervision, JT and SP; project administration, AS, JT, and SP; funding acquisition, AS, JT, and SP. All authors have read and agreed to the published version of the manuscript.

## Funding

JT was funded by grants GOC2319N, GOA4919N, and G0E7120N (F.W.O. Vlaanderen). SP was supported by KU Leuven funding (PDM/19/164) and grant 12W7822N (F.W.O. Vlaanderen). AS was supported by SB F.W.O Vlaanderen funding (1S51521NI).

## Acknowledgments

We thank Arne Bonneure and Lieze Wolput (Toxicology & Pharmacology, KU Leuven) for the technical assistance with Q-TOF-MS experiments.

## Conflict of interest

The authors declare that the research was conducted in the absence of any commercial or financial relationships that could be construed as a potential conflict of interest.

## Publisher’s note

All claims expressed in this article are solely those of the authors and do not necessarily represent those of their affiliated organizations, or those of the publisher, the editors and the reviewers. Any product that may be evaluated in this article, or claim that may be made by its manufacturer, is not guaranteed or endorsed by the publisher.
