# Supplementary material for: Unraveling the venom chemistry with evidence for histamine as key regulator in the envenomation by caterpillar Automeris zaruma
Source: Front Immunol. 2022 Aug 24;13:972442. doi: 10.3389/fimmu.2022.972442 (PMC9448982; doi:10.3389/fimmu.2022.972442)
Supplement: Supplementary file 1 [file DataSheet_1.pdf]

# Supplementary Material

## 1 Supplementary Figures

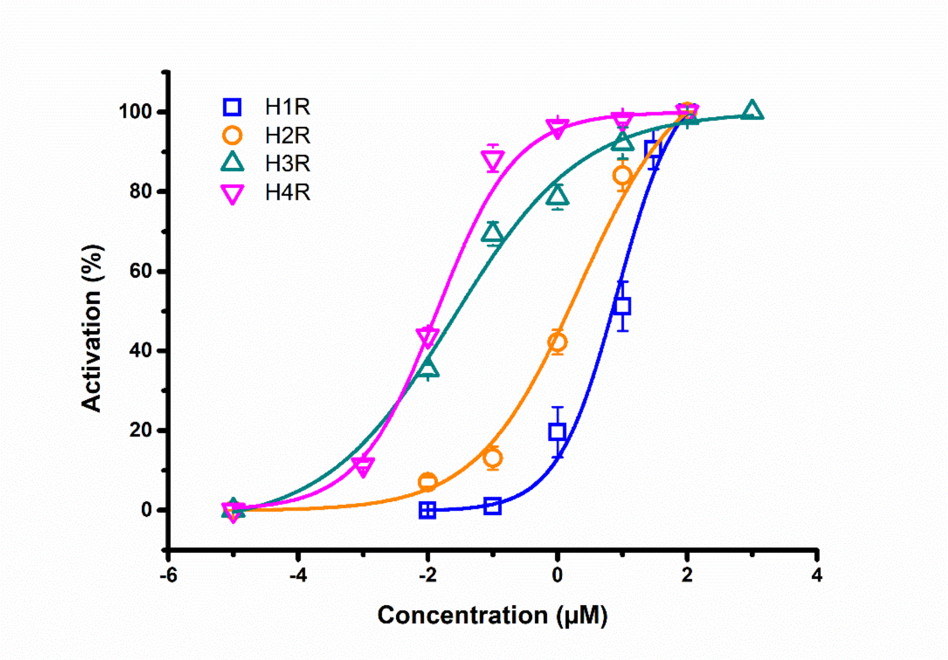


**Supplementary Figure S1.** Activation-response curve of H1R, H2R, H3R and H4R. The percentage of activation of histamine in **(blue)** H1R, **(orange)** H2R, **(green)** H3R, or **(pink)** H4R was plotted against the logarithm of the different concentrations tested and fitted with the Hill equation. The corresponding $EC_{50}$ value for H1R, H2R, H3R and H4R yielded 8 ± 3 μM, 2 ± 1 μM, 24 ± 1 nM and 13 ± 1 nM histamine respectively. The visualized error bars represent the standard error of the mean (S.E.M). All experiments were repeated at least three times (n ≥ 3).

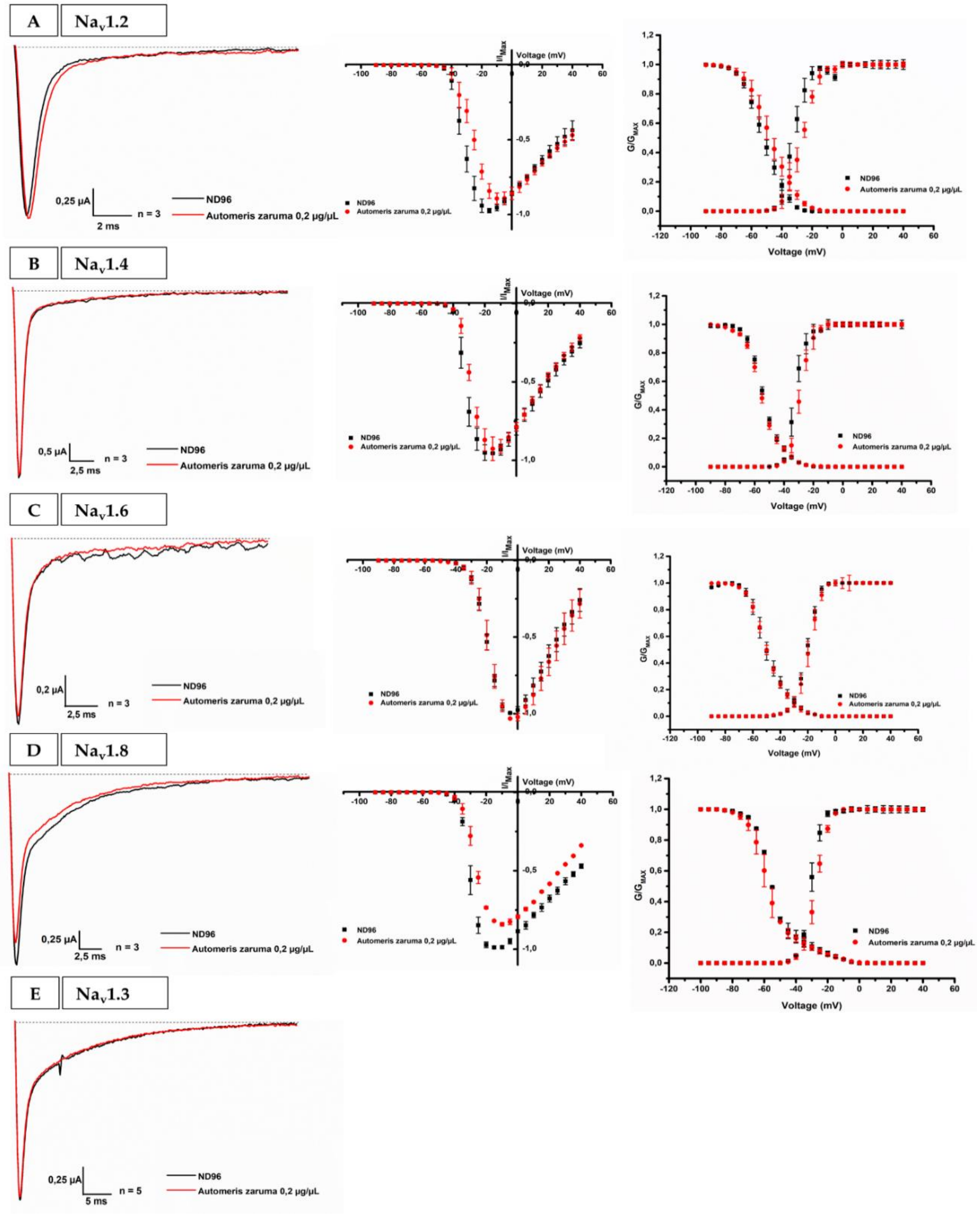


**Supplementary Figure S2.** Electrophysiological characterization of *A. zaruma* on mammalian $Na_v$ Channels. The left and the middle panels show the current voltage relationship and the right panel represent the steady-state activation and inaction curves in control ND96 buffer (black) and toxin condition 0.2 µg/µL *A. zaruma* venom extract (red) for (A) $Na_v1.2$, (B) $Na_v1.4$, (C) $Na_v1.6$, (D) $Na_v1.8$ and (E) $Na_v1.3$. All experiments were repeated at least three times ($n \geq 3$).

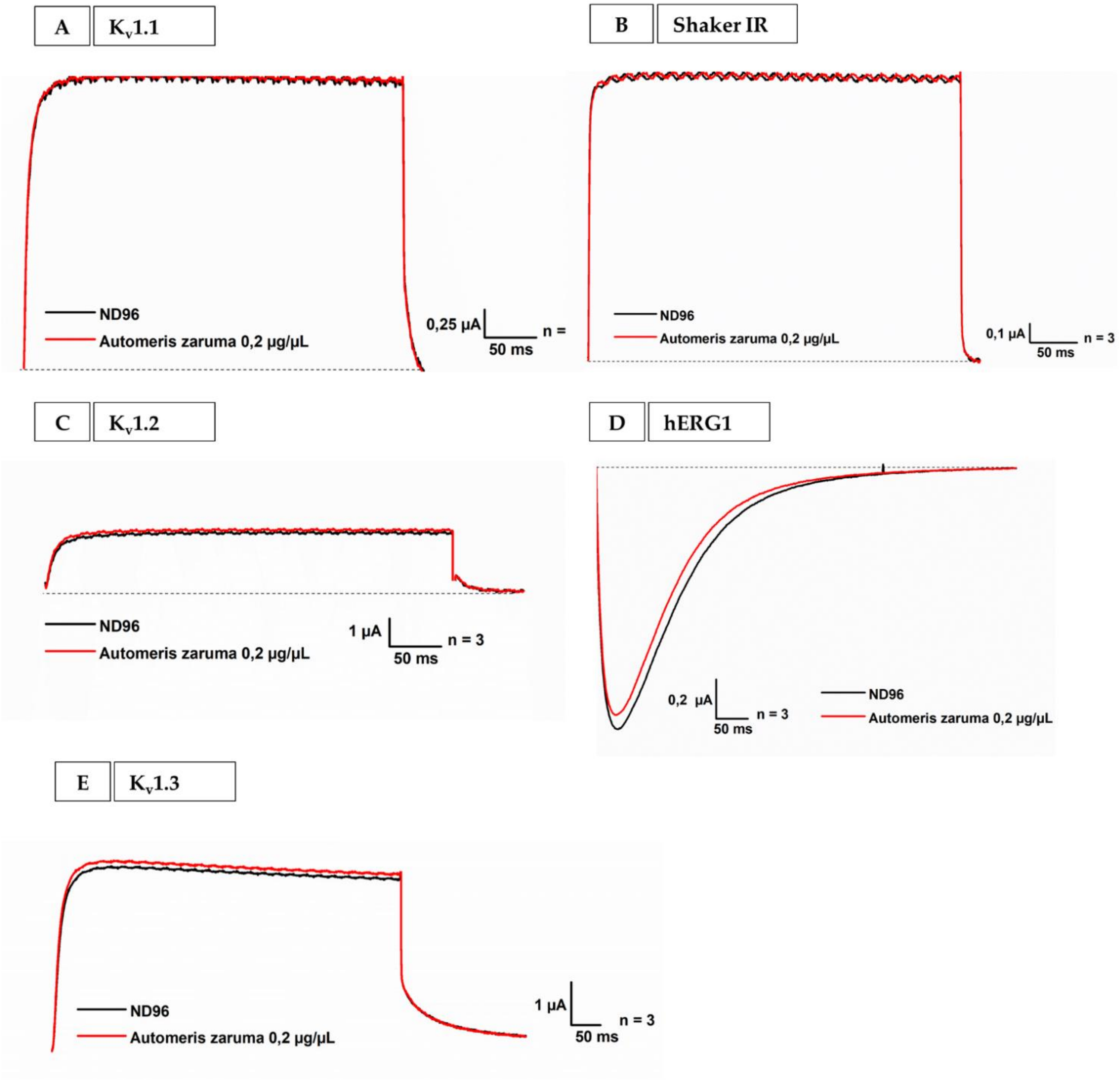


**Supplementary Figure S3.** Electrophysiological activities of *A. zaruma* on different voltage-gated potassium channels. The black lines represent the control ND96 buffer condition and the red line indicate the currents obtained in the presence of 0.2 µg/µL *A. zaruma* venom extract for (A) $K_V1.1$, (B) Shaker IR, (C) $K_V1.2$, (D) hERG1 and (E) $K_V1.3$. All experiments were repeated at least three times ($n \geq 3$).

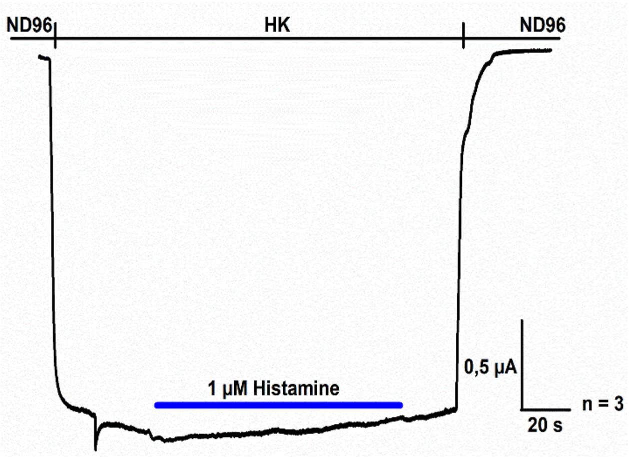


**Supplementary Figure S4.** Electrophysiological activity of histamine on oocytes co-expressing MRGPRX2 and GIRK1 and GIRK2. No current enhancement in the presence of 1 µM histamine (blue). All experiments were repeated three times ($n \geq 3$).

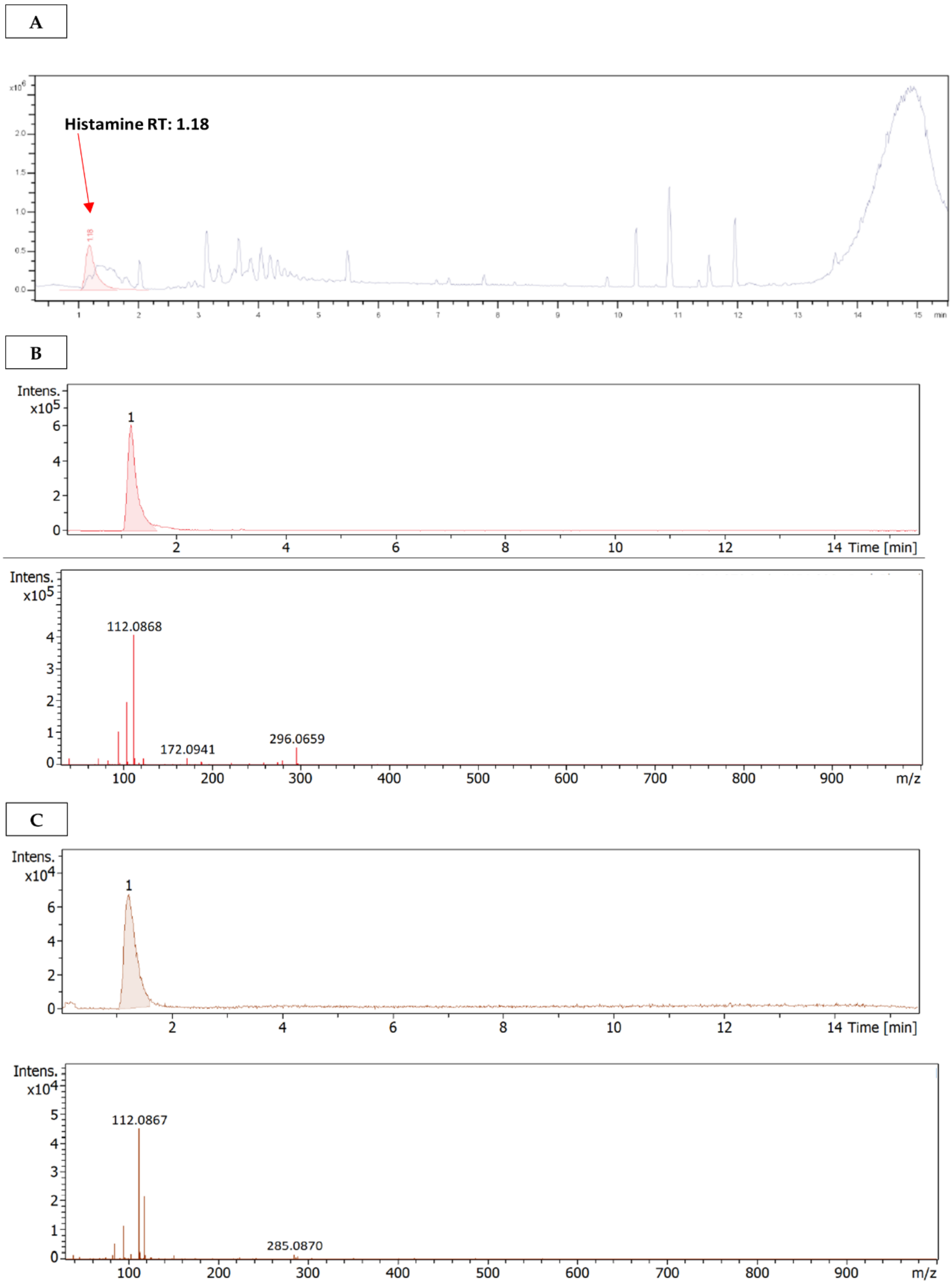


**Supplementary Figure S5**. Discovery of histamine in the venom extract of *A. zaruma* via Q-TOF-MS. (A) LC-MS chromatogram of *A. zaruma* venom extract (B) above: extracted ion chromatogram of *A. zaruma* venom at selected ion m/z = 112.0868 (histamine), below: MS/MS spectrum obtained by fragmentation of the ion m/z = 112.0868 (C) above: extracted ion chromatogram of 10 µM histamine at selected ion m/z = 112.0867 (histamine), below: MS/MS spectrum obtained by fragmentation of the ion m/z = 112.0867.

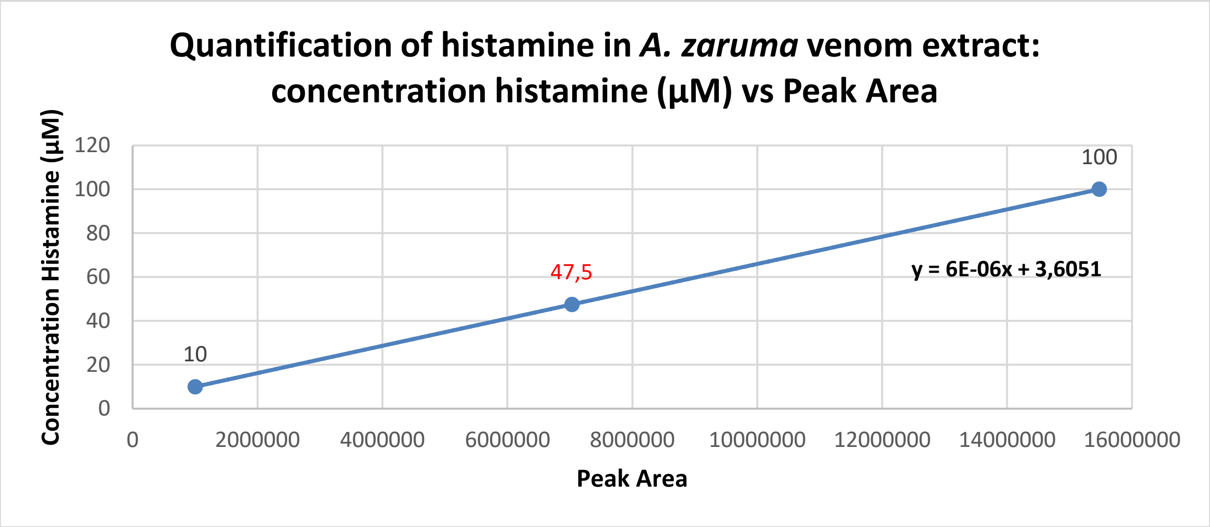


**Supplementary Figure S6.** Quantification of histamine in *A. zaruma* venom extract. Calibration curve represented as concentration of histamine in µM plotted versus the determined peak area. The determined peak area of histamine in *A. zaruma* extract was 7031600. This correspond to a concentration of 47,5 µM in a stock-solution of 10 µg/µL.
